# The impact of diabetes and social, biologic and behavioral determinants of health on liver cancer risk

**DOI:** 10.3389/fendo.2025.1562854

**Published:** 2025-06-27

**Authors:** Marwa Shouman, Ayad A. Jaffa, Miran A. Jaffa

**Affiliations:** ^1^ Epidemiology and Population Health Department, Faculty of Health Sciences, American University of Beirut, Beirut, Lebanon; ^2^ Department of Biochemistry and Molecular Genetics, Faculty of Medicine, American University of Beirut, Beirut, Lebanon

**Keywords:** Behavioral Risk Factor Surveillance System (BRFSS) 2022, behavioral, biologic and social determinants of health, diabetes, liver cancer, weighted logistic regression analysis

## Abstract

**Background:**

Liver cancer has seen a concerning rise in incidence, currently ranked as the sixth most prevalent cancer. Diabetes, along with indices of social, biological, and behavioral determinants of health, was linked to the risk of liver cancer.

**Aim:**

We aim to determine the effect of diabetes and selected indices of determinants of health on liver cancer.

**Methods:**

Our quantitative study is based on a sample of 239,006 US participants adopted from the BRFSS-2022 data. Our results are summarized using frequency distributions and weighted percentages. Weighted logistic regressions were employed to determine the associations with liver cancer.

**Results:**

In this sample population, 73 individuals experienced liver cancer, 12.17% (n=33,776) had diabetes, with a four-fold increase in the odds of liver cancer for individuals with diabetes (OR: 4.27, CI: 1.73-10.57). Employment status, educational level, urban/rural living, smoking status were determinants of health associated with liver cancer as well. Our subgroup analysis focusing exclusively on those diagnosed with liver cancer following their diabetes diagnosis confirmed diabetes as risk factor for liver cancer (OR: 5.44, 95%CI: 1.58-18.70), along with marital status and other determinants of health.

**Conclusion:**

Effective diabetes management and addressing key health determinants are crucial for reducing liver cancer risk and improving prevention and treatment outcomes.

## 1 Introduction

Primary liver cancer is the sixth most common cancer and the third leading cause of cancer-related deaths (1). It arises from malignant tumor cells forming in liver tissues (1). Liver cancer can be classified into several types based on the site of its origin. Hepatocellular Carcinoma (HCC) is the predominant type of liver cancer originating in hepatocytes and accounts for about 80 to 90% of liver cancer cases (2). Cholangiocarcinoma, stemming from bile duct cells, accounts for about 10 to 20% of liver cancer cases, while angiosarcoma, a rare type, constitutes less than 2% of liver cancer cases, and originates in blood vessels, mainly affects adults over 70 years of age (3).

Cirrhosis of the liver is an essential risk factor associated with liver cancer. Cirrhosis develops when scar tissues replace the damaged liver cells. Individuals who suffer from cirrhosis face heightened susceptibility to liver cancer. Remarkably, the majority of liver cancer cases, up to 90% in the States (US), stem from pre-existing cirrhosis (4, 5).

The incidence of liver cancer has been increasing over the past several years, rising from 2.641 cases per 100,000 person-years in 1975 to 8.657 cases per 100,000 person-years in 2017 (6). In 2020, around 905,677 individuals were diagnosed with liver cancer, globally, marking an age-standardized incidence rate of 9.5 per 100,000 individuals (7).

Social determinants of health (SDOH), defined as the social and economic factors that shape health outcomes, are known to be associated with liver cancer risk. For instance, race is a significant risk factor for liver cancer, with its incidence varying across different racial groups (8). African Americans have a higher incidence rate ratio (1.4) compared to White Americans (8), and are more likely to develop advanced liver cancers with less advanced liver disease (9). Moreover, according to the OHSU Knight Cancer Institute, Native Americans and Native Alaskan adults are at a higher risk of developing liver cancer among other racial and ethnic groups (10). These findings underscore the role of racial and ethnic disparities, along with broader social determinants of health (SDOHs) in influencing liver cancer risk. A recent study in the European Economic Area and the United Kingdom concluded that individuals with low socioeconomic status (SES), who reside in disadvantaged areas with low educational levels, face elevated death rates from cirrhosis and context-related increased risk of liver cancer (11). Particularly, they may face barriers such as discrimination and inadequate healthcare access in liver cancer care (11), highlighting the impact of socioeconomic factors on liver cancer risk.

Employment and occupational types can also affect the risk of certain types of cancer. In this regard, a recent study, included over 12,000 individuals analyzing their employment history from age 16 to 65, revealed that certain employment trajectories were associated with varying cancer risks and suggested that employment history can influence cancer development (12). In addition, certain occupations and workplace exposures were found to be associated with an increased risk of liver cancer that are due to contact with specific chemicals and toxins (13–15). Aflatoxin, for example, accounts for approximately 20% of liver cancer cases worldwide, with a high proportion occurring in sub-Saharan Africa, mainly among farmers and textile workers engaged in agricultural activities (14), whereas exposure to endotoxin may be protective against liver cancer risk (16), underscoring the impact of socioeconomic and occupational factors on liver cancer risk.

Interestingly, liver cancer prognosis has also been shown to be affected by partner status, with patients who are married or with partners having positive prognosis and better survival rates compared to single, unmarried or widowed participants (17–19). This link may be explained by the emotional and social support a person living with cancer can receive from a partner which can help alleviate the psychological distress triggered by their cancer diagnosis (20–22). This suggests that marital status, a key index of social determinant of health, may also play a role in influencing liver cancer risk.

Certain biologic characteristics were also identified as potential correlates of liver cancer. In this respect, it was reported that the age-standardized incidence rate for liver cancer is 14.1 per 100,000 for males and 5.2 per 100,000 for females (23). According to estimates by the American Cancer Society for 2023, primary liver cancer and intrahepatic Cholangiocarcinoma (ICC) are projected to affect approximately 41,630 individuals in the US. Among these cases, about 28,000 are expected to affect men and 13,630 to affect women (5), showing that men are at a higher risk of developing liver cancer. A recent study in China concluded that about 45% of liver cancer cases were among adults aged 60–79 years and 37% were among 45–59 years adults suggesting that comorbidities associated with aging, and older ages elevate liver cancer risk (23). Furthermore, obesity, classified as having Body Mass Index (BMI) >35 Kg/m^2^ is also identified as a risk factor for liver cancer (24–26). For instance, there is a 39% higher risk of HCC for every 5-unit rise in BMI in a meta-analysis of 21 prospective studies that included 17,624 cases of primary liver cancer (27). Moreover, a recent systematic review and meta-analysis of 28 prospective cohort studies concluded that an increased BMI, particularly obesity, is associated with a three times higher risk of developing primary liver cancer (28). These findings highlight the role of biological factors, such as age, gender, and BMI, on the risk of this type of malignancy.

Behavioral and lifestyle factors such as alcohol consumption, smoking, and exercise can also affect liver cancer. For instance, findings from the World Health Organization’s International Agency for Research on Cancer (WHO-IARC) have established alcohol consumption as a causal factor in the development of liver cancer (29), accounting for 32% of HCC cases in Italy (30) and 45% in the US (31). A recent meta-analysis further confirmed these findings, indicating that heavy alcohol consumption is associated with double the risk of developing two specific types of liver cancer: HCC and ICC (32). Additionally, the Liver Cancer Pooling Project, consisting of 14 cohort studies in the US, revealed an 87% increased risk of HCC among heavy alcohol drinkers compared to non-drinkers (33). Furthermore, several studies confirmed that liver cancer risk decreases by 6% to 7% after cessation of alcohol consumption, emphasizing the significant impact of lifestyle and behavioral factors on liver cancer risk (31). In addition, alcohol consumption is a recognized risk factor for liver cirrhosis, with a dose-response relationship (34, 35). A prospective study of 401,806 women in the UK, conducted over 15 years of follow-up, revealed that the incidence of liver cirrhosis increases with higher alcohol intake. Compared to consuming 1 or 2 drinks per week, drinking 15 or more drinks weekly increases the relative risk of cirrhosis nearly 3 times (35). Along the same lines, studies have highlighted an elevated risk of HCC among current smokers (33, 36), with a smoker who consumes more than 25 cigarettes per day facing a 55% increased risk of HCC (33). Furthermore, in recent studies, exercise interventions have been linked to improving health parameters for liver cancer patients (37), and liver cirrhosis patients significantly (38). A meta-analysis of 10 prospective cohort studies concluded that engaging in vigorous-intensity physical exercise in high amounts reduces liver cancer risk by 54% (39). These findings underscore the importance of lifestyle choices on the liver cancer risk.

In addition to the biological, social and behavioral factors, diabetes is a comorbid disease that also contributes to elevated liver cancer risk (40). Diabetes is a highly prevalent chronic disease that affects about 37 million Americans, including youth and adults (41). Individuals with type 2 diabetes face a heightened 2 to 4 times risk of developing liver cancer compared to non-diabetics, suggesting an increased susceptibility to severe liver diseases such as HCC in diabetic patients (42, 43). Furthermore, a study conducted among patients with cirrhosis found that, in the multivariable analysis, diabetes was associated with a significantly increased risk of HCC, with a hazard ratio of 4.2 (44). The increased incidence and the advancement of chronic liver disease among individuals with diabetes may be linked to an increased risk of liver cancer, with several factors involved, such as viral infections and excessive alcohol consumption (40). In addition, diabetes is strongly influenced by various social determinants of health (SDOHs), socio-demographic factors, and behavioral patterns (45–47). Therefore, it is essential to examine the association between diabetes and liver cancer within the context of these diverse variables.

Due to the rising incidence of liver cancer cases, it’s critical to identify and address the causes of this increase. Several factors, such as diabetes and other social, biological, and behavioral variables, fall within our sphere of influence. However, current research lacks a comprehensive understanding of all the contributing factors. That’s where our study comes in: we seek to fill this gap by investigating how diabetes, biological, social health determinants, and behavioral choices can influence liver cancer risk. In this study, we aim to explore how various indices of social determinants of health (SDOH), including race, employment, education, marital status, and others, are correlated with liver cancer, alongside behavioral factors such as smoking, alcohol consumption, and exercise, and biological factors encompassing age, sex, and BMI. The novelty of our study lies in the inclusion of diabetes, a highly prevalent clinical condition, alongside a wide array of non-clinical characteristics to assess their relationships comprehensively with this specific type of malignancy.

## 2 Methods

### 2.1 Study population and sampling

**Figure 1:**
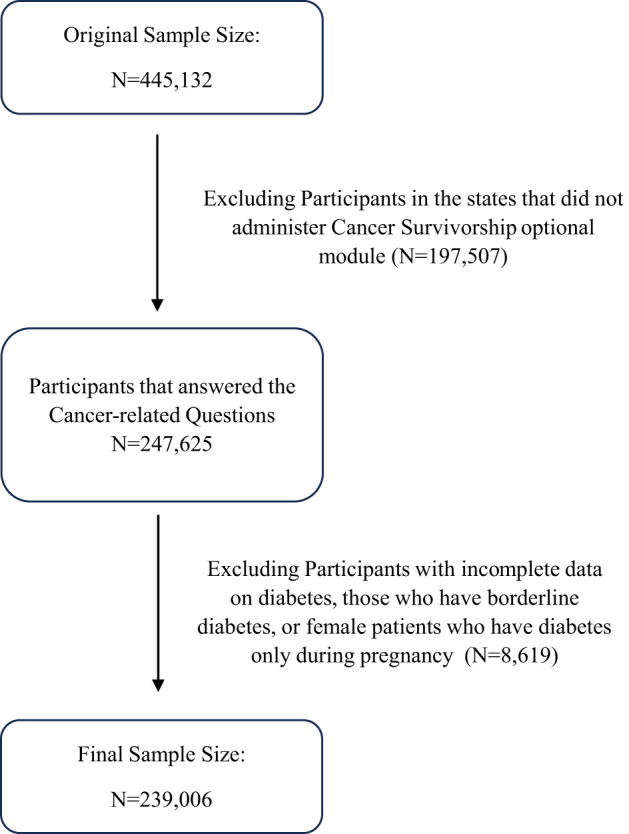
Flowchart illustrating how the final sample size was reached.

Our quantitative study is based on secondary data retrieved from the Behavioral Risk Factor Surveillance System (BRFSS) collected cross-sectionally in the year 2022. The BRFSS, administered by the Centers for Disease Control and Prevention (CDC), serves as the primary health-related telephone survey system in the US. It aims to gather state-specific data on various health-related risk behaviors, chronic health conditions, and the use of preventive services among U.S. residents. This survey system included noninstitutionalized adults aged 18 years or older residing in 50 US states and participating regions, totaling 445,132 participants. Not all states administered the “Cancer Survivorship: type of cancer” optional module; accordingly, 30 states were included in this study. After excluding states without responses to cancer-related questions, the sample size decreased to 247,625 individuals. Diabetes serves as the primary predictor in our study, so it is important to exclude participants with incomplete data on diabetes. As a result, the final sample size for our analysis is 239,006 participants, presented in the flowchart depicted in **Figure 1**.

The BRFSS selects participants randomly for interviews, using a multistage cluster design, resulting in a nationally representative sample. Aiming to adequately represent smaller and geographically distinct regions of interest, a weighted sampling approach was used to collect data disproportionately.

### 2.2 Sub-analysis study population

To ensure that liver cancer occurred after diabetes, we excluded participants whose diabetes was diagnosed after their liver cancer. Specifically, we focused on participants with only one type of cancer, liver cancer, and created a new variable representing the age difference between the diagnosis of cancer and diabetes (cancer diagnosis age - diabetes diagnosis age). Participants with a negative age difference, indicating that cancer was diagnosed before diabetes, were excluded from the study. As a result, 42 observations were removed, and the final sample size is 238,964 for this sub-analysis.

### 2.3 Concepts and measures

The outcome of interest in our study is liver cancer which was classified as a binary yes-no question. Participants who reported being diagnosed with cancer were further asked about the specific type they had, and those indicating liver cancer were considered the cases in our study.

Diabetes, the primary predictor in our quantitative analysis, was dichotomously classified based on participants’ self-reported diabetes status. Other predictors, encompassing SDOHs, include variables such as race dichotomized into white and non-white, home ownership, marital status, employment status, income level, place of residence, educational level, and race.

In addition, several biological and behavioral determinants of health were also considered in our analysis. These factors included age, sex, body mass index (BMI), exercise, smoking status, and alcohol consumption. Participants who reported consuming at least one alcoholic drink in the past 30 days were categorized as alcohol drinkers and assigned a value of “Yes” for the alcohol use variable. BMI was categorized according to the CDC classification into four groups: underweight (BMI less than 18.5 kg/m²), normal weight (BMI between 18.5 kg/m² and 25.0 kg/m²), overweight (BMI between 25.0 kg/m² and 30.0 kg/m²), and obese (BMI more than 30.0 kg/m²). Data on smoking were collected using two questions. First, participants were asked, “Have you smoked at least 100 cigarettes in your entire life?” Those who answered “No” were classified as never smokers, while those who answered “Yes” were further categorized. To distinguish between former and current smokers, a follow-up question was asked: “Do you now smoke cigarettes every day, some days, or not at all?” Participants who answered every day or some days were classified as current smokers, while those who answered not at all were classified as former smokers.

Further details about the distribution and categories of our variables can be found in the descriptive table (**Table 1**).

**Table 1:** Characteristics of the study population.

Variables	N	Weighted %
Outcome		
Liver Cancer		
Yes	73	2.7*10^-4^
No	198,695	99.97
Predictors		
Diabetes		
Yes	33,776	12.17
No	205,230	87.83
Social Determinants of Health		
Race		
Other race^2^	58,378	35.24
White	173,571	64.76
Home Ownership		
Own	167,467	69.88
Rent	57,546	23.7
Other Arrangement	11,619	6.42
Marital Status		
Married or have a partner	133,250	55.42
Divorced or Separated	35,024	12.68
Widowed	25,444	7.29
Never Married	42,903	24.61
Employment Status		
Employed or Self-Employed	123,411	58.66
Unemployed ^1^	24,177	14.15
Retired	72,098	21.16
Unable to work	13,458	6.03
Income Level		
Less than $15,000	11,321	6.62
$15,000 to 34,000	40,932	21.87
$35,000 to 74,000	57,247	29.19
$75,000 to $150,000	53,846	28.24
More than $150,000	24,730	14.09
Social Determinants of Health	N	Weighted %
Place of Residence		
Urban	208,347	93.15
Rural	25,490	6.85
Education Level		
Some high school or below	13,347	10.08
high school graduate	59,157	28.06
some college or technical school	64,386	30.62
College graduate	101,022	31.24
Biological and Behavioral Variables		
Age		
18 to 34	40,825	29.19
35 to 54	68,546	31.38
55 to 64	44,408	16.18
65 or older	85,227	23.26
Sex		
Male	113,285	49.24
Female	125,721	50.76
Body-Mass Index (BMI)		
Normal Weight	63,456	30.52
Underweight	3,643	1.97
Overweight	75,726	34.31
Obese	70,616	33.2
Smoking Status		
Never Smoked	133,397	62.3
Current Smoker	26,654	13.22
Former Smoker	60,480	24.48
Alcohol Consumption		
Yes	113,420	53.43
No	101,010	46.57
Exercise		
Yes	181,446	76.44
No	57,054	23.56

^1^Unemployed included homemakers, students, those who are out of work for 1 year or more, and those who are out of work for less than 1 year. ^2^Other race included Black, Asian, Multiracial, Hispanic American Indian or Alaskan Native and Native Hawaiian or other Pacific Islander.

### 2.4 Statistical analysis

Accounting for the complex sampling weighted approach in the BRFSS data collection process, our analysis required weighted descriptive and regression analyses. Our analysis was initiated by generating summary statistics, including counts and weighted percentages reported for all variables in the study to determine the distribution of the different levels of the variables among participants. The crude associations were evaluated using weighted simple logistic regression computing unadjusted odds ratios (ORs) along with their 95% confidence intervals (CIs) for the associations between diabetes, SDOH, biologic and behavioral factors, and liver cancer. A p-value that is less than or equal to 0.05 reveals a significant predictor for liver cancer risk. Subsequently, we built our multivariable model using weighted multiple logistic regression, and including most of the variables that were significant at the unadjusted level, yielding adjusted ORs along with their 95% CIs, and p-values. Our multivariable models were built gradually by adding each of the covariates one at a time while monitoring the changes in the covariates’ magnitude and direction. This gradual approach in model building helped us avoid issues of multicollinearity among predictors whilst ensuring that measures of multicollinearity such as the variance inflation factor remained low and below the conservative cutoff point of 2.5 assumed for logistic regression. We have employed the statistical package StataSE 18 for analysis of the data.

## 3 Results

### 3.1 Descriptive table

The characteristics of our study population are summarized in **Table 1**. The total number of participants in our study is 239,006, among whom 73 individuals experienced the outcome of interest, liver cancer. Diabetes is prevalent among 12.17% of the participants (n=33,776).

The majority of participants in our study reside in urban areas (93.15%, n=208,347), own homes (69.88%, n=167,467), and are either married or have a partner (55.42%, n=144,250). A balanced distribution of income is observed, with 21.87% of the population earning between $15,000 and $34,000, 29.19% between $35,000 and $74,000, and around 28.24% between $75,000 and $150,000. A small percentage (6%) have income levels below $15,000, while 14% have incomes exceeding $150,000.

Most of the participants are employed or self-employed (58.66%, n=123,411), while 14.15% (n=24,177) are unemployed, including homemakers, students, and those out of work. Educational levels vary, with around 10% having some high school education or below. The rest are distributed among high school graduates (28.06%), those with some college or technical school attendance (30.62%), and college graduates (31.24%).

Regarding race, the majority are identified as white (64.76%, n=173,571), with the remaining participants falling into the “other race” category, including Black, Asian, Multiracial, Hispanic, American Indian or Alaskan Native, and Native Hawaiian or other Pacific Islander. Gender distribution is nearly equal, with 50.76% females (n=125,721) and 49.24% males (n=113,285).

Concerning the biological factors, around 60% of participants fall within the younger age group of 18 to 54 (n=109,371), while approximately 40% (n=129,635) constitutes the older age group of 55 years and above.

BMI categories, particularly normal weight, overweight, and obese, exhibit a relatively even distribution, each comprising approximately one-third of the study cohort, with underweight participants accounting for a minimal prevalence of about 2%. In terms of behavioral factors, the majority of participants reported never smoking (62.3%, n=133,397), engaging in regular physical exercise (76.44%, n=181,446), and consuming alcohol in the 30 days before data collection (53.43%, n=113,420).

### 3.2 Liver cancer, diabetes, and SDOHs: unadjusted and adjusted associations

**Table 2** presents the weighted unadjusted ORs of the associations between liver cancer and all the predictors considered in our study to examine the crude associations. The purpose of these associations is to identify significant predictors, defined by a p-value less than 0.05, for inclusion in the subsequent multivariable model, **Table 3**.

**Table 2:** Unadjusted associations: liver cancer, diabetes, SDOH, and other covariates.

Liver cancer	Weighted unadjusted OR (95% CI)	p-value
Diabetes		
No	Ref	
Yes	7.77 (3.41- 17.67)	<0.001*
Social Determinants of Health		
Race		
Other race^2^	Ref	
White	1.67 (0.52-5.44)	0.394
Home Ownership		
Own	Ref	
Rent	0.37 (0.13-1.06)	0.065
Other Arrangement	1.00 (0.29-3.46)	0.988
Marital Status		
Married or Coupled	Ref	
Divorced or Separated	3.40 (1.45-7.93)	0.005*
Widowed	3.66 (0.65-20.43)	0.139
Never Married	0.66 (0.25-1.74)	0.409
Employment Status		
Employed or Self-Employed	Ref	
Unemployed ^1^	0.73(0.095-5.69)	0.771
Retired	8.06 (3.19-20.36)	<0.001
Unable to work	7.4 (2.33-23.54)	0.001*
Place of Residence		
Rural	Ref	
Urban	0.19 (0.06-0.62)	0.006*
Education Level		
Some high school or below	Ref	
high school graduate	4.68 (1.56-14.01)	0.006*
some college or technical school	0.99 (0.34-2.90)	0.988
College graduate	1.81 (0.57-5.69)	0.309
Income Level		
Less than $15,000	Ref	
$15,000 to $34,000	0.73 (0.18-2.92)	0.661
$35,000 to $74,000	0.22 (0.06-0.84)	0.027*
$75,000 to $150,000	0.18 (0.037-0.88)	0.034*
More than $150,000	0.22 (0.036-1.38)	0.108
Biological and Behavioral Variables		
Age		
18 to 34	Ref	
35 to 54	1.70 (0.28-10.36)	0.562
55 to 64	10.11 (1.87-54.58)	0.007*
65 or older	16.97 (3.31-87.03)	0.001*
Sex		
Male	Ref	
Female	1.01 (0.44-2.29)	0.969
Body-Mass Index (BMI)		
Normal Weight	Ref	
Underweight	3.02 (0.54-16.87)	0.207
Overweight	0.74 (0.27-2.01)	0.562
Obese	1.26 (0.45-3.47)	0.651
Smoking Status		
Never Smoked	Ref	
Current Smoker	5.02 (1.50-16.78)	0.009*
Former Smoker	4.53 (2.22-9.25)	<0.001*
Alcohol Consumption		
No	Ref	
Yes	0.33 (0.107-1.03)	0.057
Exercise		
No	Ref	
Yes	0.77 (0.34-1.75)	0.54

^1^Unemployed included homemakers, students, those who are out of work for 1 year or more, and those who are out of work for less than 1 year.

^2^Other race included Black, Asian, Multiracial, Hispanic American Indian or Alaskan Native and Native Hawaiian or other Pacific Islander.

*p-value ≤ 0.05 indicating significant results.

**Table 3:** Adjusted associations: liver cancer, diabetes, SDOH, and other covariates.

Liver cancer	Weighted adjusted OR (95% CI)	p-value
Diabetes		
No	Ref	
Yes	4.27 (1.73-10.57)	0.002*
Social Determinants of Health		
Marital Status		
Married or Coupled	Ref	
Divorced or Separated	2.28 (0.96-5.41)	0.061
Widowed	1.54 (0.31-7.47)	0.592
Never Married	1.37 (0.53-3.51)	0.506
Employment Status		
Employed or Self-Employed	Ref	
Unemployed ^1^	0.01(0.002-0.13)	<0.001*
Retired	4.50 (1.75-11.58)	0.002*
Unable to work	3.33 (0.84-13.17)	0.085
Education Level		
Some high school or below	Ref	
high school graduate	7.04 (1.94-25.51)	0.003*
some college or technical school	1.80 (0.52-6.17)	0.346
College graduate	5.09 (1.24-20.83)	0.024*
Place of Residence		
Rural	Ref	
Urban	0.24 (0.08-0.71)	0.01*
Behavioral factors		
Smoking Status		
Never Smoked	Ref	
Current Smoker	3.05 (0.79-11.68)	0.103
Former Smoker	2.55 (1.20-5.42)	0.015*

^1^Unemployed included homemakers, students, those who are out of work for 1 year or more, and those who are out of work for less than 1 year.

*p-value ≤ 0.05 indicating significant results.

Results from the unadjusted analysis indicate that diabetes, marital status, employment status, place of residence, educational level, income level representing indices of SDOH, along with age and smoking which represent the biologic and behavioral determinants of heath respectively are significantly associated with liver cancer risk. As such, some of these variables were incorporated in **Table 3** to construct the multivariable model. Recognizing that both income and employment status are components of SDOH and are correlated with financial status, and noting that employment status can also be affected by age, combining all these covariates together in one model may not be feasible given their strong interconnectivity. Consequently, employment status was included in our multivariable model excluding income and age.

The findings of the full model underscore a significant association between diabetes and liver cancer, indicating a four-fold increase in the odds of liver cancer for individuals with versus without diabetes (OR: 4.27, CI: 1.73-10.57, p-value: 0.002) (**Table 3**).

With respect to the SDOH, marital status, initially significant in the unadjusted analysis, had an increase in p-value and became borderline significant for the divorced/separated category compared to the married or coupled participants (OR: 2.28, CI: (0.96-5.41, p-value: 0.061). However, employment status emerges as a crucial factor, with unemployment showing a protective association with liver cancer (OR: 0.01; 95% CI: 0.002-0.13, p-value: 0.000). Conversely, being retired or unable to work appears to be linked with increased odds of liver cancer by approximately three and four times, respectively, compared to employed individuals. Educational level had a significant association with liver cancer, with high school graduates exhibiting a seven-fold increase in the odds of liver cancer compared to those with some schooling or below (OR: 7.04, 95% CI: 1.94-25.51, p-value: 0.003). Living in an urban area emerges as a protective factor, that is significantly associated with reduced risk of liver cancer (OR: 0.24, 95% CI: 0.08-0.71, p-value: 0.01).

Regarding the behavioral factors, smoking status was significantly associated with liver cancer in the full model indicating that former smokers had twice the odds of liver cancer compared to those who never smoked (OR: 2.55, 95% CI: 1.20-5.42, p-value: 0.015) (**Table 3**).

### 3.3 Sub-analysis: characteristics of the participants with liver cancer diagnosed after preexisting diabetes and the weighted adjusted associations with diabetes, and the other variables in this subpopulation (n=238,964)

After excluding participants diagnosed with diabetes after their liver cancer, the number of liver cancer cases decreased to 31 within the subpopulation of 238,964. In this group, the prevalence of diabetes is 12.16% (n=33,754) (**Table 4**). Similar to our initial approach, we conducted unadjusted analyses to identify significant variables and build our new model. Diabetes, marital status, employment status, place of residence, and smoking status were the variables included in our adjusted model of the sub-group. Our adjusted sub-analysis presented in **Table 5** showed a 5-fold increase in liver cancer risk for diabetics (OR: 5.44; 95% CI (1.58-18.70)), compared to non-diabetics. Moreover, employment status was also associated with liver cancer among participants who had diabetes before liver cancer. Specifically, compared to employed participants, those who were retired had a higher risk of liver cancer (OR: 3.30; 95% CI: 1.00-10.86), while unemployed participants appeared to be protected against liver cancer (OR: 0.02; 95% CI: 0.002-0.18). Marital status was also linked to liver cancer. In particular, widowed participants had a six-fold higher risk (OR: 6.37; 95% CI: 1.17-34.58), while those who never married had a four-fold higher risk (OR: 4.24; 95% CI: 1.14-15.72) compared to participants who were married or in a couple. Lastly, place of resident was also associated with liver cancer risk whereby residing in urban areas was linked to an 84% reduced risk (OR:0.16; 95%CI (0.032-0.81)). These results confirm once more diabetes and social determinants of health as significant associates of liver cancer.

**Table 4:** Characteristics of the participants with liver cancer diagnosed after preexisting diabetes and the weighted unadjusted associations with diabetes, and the other variables in this subpopulation.

Total sample size (238,964)				
Variables	N	Weighted %	Weighted unadjusted OR (95% CI)	P-value
Outcome				
Liver Cancer				
Yes	31	1.3*10^-4^		
No	198,695	99.99		
Predictors				
Diabetes				
Yes	33,754	12.16	Ref	
No	205,210	87.84	7.85 (2.03-30.26 )	0.003*
Social Determinants of Health				
Race				
Other race^2^	58,376	35.24	Ref	
White	173,532	64.76	0.65 (0.16-2.55)	0.538
Home Ownership				
Own	167,435	69.88	Ref	
Rent	57,540	23.7	0.79 (0.20-3.15)	0.749
Other Arrangement	11,616	6.42	1.59 (0.25-9.83)	0.618
Marital Status				
Married or coupled	133,226	55.42	Ref	
Divorced or Separated	35,013	12.68	5.42 (1.53-19.10)	0.009*
Widowed	25,441	7.29	12.93 (1.88-88.86)	0.009*
Never Married	42,899	24.61	1.85 (0.51-6.72)	0.349
Employment Status				
Employed or Self-Employed	123,403	58.66	Ref	
Unemployed ^1^	24,177	14.15	1.29 (0.15-10.97)	0.812
Retired	72,073	21.16	6.59 (1.52-28.51)	0.012*
Unable to work	13,449	6.03	3.07 (0.58-16.06)	0.183
Place of Residence				
Rural	25,477	6.84	Ref	
Urban	208,319	93.16	0.13 (0.02-0.88)	0.037*
Social Determinants of Health				
Income Level				
Less than $15,000	11,318	6.61	Ref	
$15,000 to 34,000	40,922	21.87	0.73 (0.18-2.92)	0.661
$35,000 to 74,000	57,239	29.19	0.22 (0.06-0.84)	0.027*
More than $75,000	78,576	42.32	0.19 (0.047-0.80)	0.024*
Biological and Behavioral Variables				
Age				
18 to 34	40,825	29.19	Ref	
35 to 54	68,542	31.38	1.21 (0.16-8.74)	0.849
55 to 64	44,396	16.18	2.27 (0.28-18.04)	0.439
65 or older	85,201	23.25	8.84 (1.44-54.16)	0.018*
Sex				
Male	113,258	49.24	Ref	
Female	125,706	50.76	1.99 (0.62-6.39)	0.246
Body-Mass Index (BMI)				
Normal Weight	63,447	30.52	Ref	
Underweight	3,642	1.97	4.98 (0.59-41.76)	0.139
Overweight	75,709	34.31	0.27 (0.07-1.02)	0.055
Obese	70,602	33.21	1.70 (0.41-7.03)	0.458
Smoking Status				
Never Smoked	133,378	62.31	Ref	
Current Smoker	26,650	13.22	5.90 (1.19-29.23)	0.030*
Former Smoker	60,461	24.47	2.13 (0.68-6.68)	0.192
Alcohol Consumption				
Yes	113,410	53.43	Ref	
No	100,979	46.57	0.60 (0.13-2.76)	0.512
Exercise				
Yes	181,420	76.44	Ref	
No	57,038	23.56	0.97 (0.27-3.49)	0.974

*p-value <= 0.05 indicating significant results.

**Table 5:** Adjusted associations: liver cancer, diabetes, SDOH, and other covariates among participants with liver cancer diagnosed after preexisting diabetes.

Total sample size (238,964), among them 31 liver cancer cases		
Liver cancer	Weighted adjusted OR (95% CI)	p-value
Diabetes		
No	Ref	
Yes	5.44 (1.58-18.70)	0.007*
Social Determinants of Health		
Marital Status		
Married or Coupled	Ref	
Divorced or Separated	3.07 (0.65-14.45)	0.154
Widowed	6.37 (1.17-34.58)	0.032*
Never Married	4.24 (1.14-15.72)	0.031*
Employment Status		
Employed or Self-Employed	Ref	
Unemployed ^1^	0.02 (0.002-0.18)	0.001*
Retired	3.30 (1.00-10.86)	0.050*
Unable to work	0.88 (0.12-6.34)	0.906
Place of Residence		
Rural	Ref	
Urban	0.16 (0.032-0.81)	0.027*
Behavioral factors		
Smoking Status		
Never Smoked	Ref	
Current Smoker	3.62 (0.51-25.75)	0.198
Former Smoker	1.12 (0.33-3.75)	0.846

*p-value <= 0.05 indicating significant results.

## 4 Discussion

We present here a study that focuses on identifying the risk factors for liver cancer using nationally representative population-based BRFSS data. We have examined diabetes in relation to liver cancer in a comprehensive model that included a broad set of SDOH, biological, and behavioral correlates of liver cancer.

Our findings indicated that diabetes, employment status, educational level, place of residence, smoking status, and marital status were significantly associated with liver cancer. Specifically, individuals with diabetes, currently employed or retired adults, high school or college graduates, residents of rural areas, former smokers, and widowed or never married, have an increased risk of liver cancer.

### 4.1 Liver cancer risk and diabetes

Previous research has identified diabetes as a risk factor for various cancer types, including liver cancer (48). Consistent with this, our study findings revealed that individuals with diabetes face a four-fold increase in the odds of liver cancer compared to those without diabetes. In a six-year cohort study consisting of 10,794 Type 2 diabetes cases, 59 primary liver cancer cases were diagnosed, indicating an incidence rate of 54.66 per 10,000 individuals (49). Similar to our results, in a hospital-based case-control study, individuals with diabetes were found to have a four times higher risk of HCC in their multivariable model (50). They also suggested that as Type 2 diabetes progresses, the risk of liver cancer increases significantly (50). Specifically, in the presence of viral hepatitis B and C and heavy alcohol intake, the risk of liver cancer was observed to increase ten times among type 2 diabetics (50), indicating that the effect of diabetes on liver cancer risk is highly impacted by other coexisting conditions. Studies showed that some metabolic conditions, such as non-alcoholic fatty liver disease (NAFLD), might mediate the associations between diabetes and the increased liver cancer risk (51, 52). NAFLD is a condition in which there is an excess fat build-up on the liver that is not related to the excess in alcohol use (53). More than 70% of individuals with diabetes have NAFLD, often due to insulin resistance (43). In individuals with NAFLD, diabetes contributes to liver cancer through complex mechanisms involving liver inflammation (54). In response to diabetes, the liver undergoes chronic inflammation characterized by the release of proinflammatory cytokines and the generation of reactive oxygen species (ROS) (54). These inflammatory mediators induce cellular stress and disrupt normal cellular homeostasis by activating intracellular signaling pathways (54). Moreover, a major consequence of diabetes-induced inflammation is the induction of genomic instability, marked by the accumulation of DNA damage and mutations within hepatocytes. This genetic instability predisposes liver cells to malignant transformation and the development of HCC (54).

Identifying diabetes as an important risk factor for liver cancer highlights the potential influence of diabetes management on reducing the risk of liver cancer. The use of metformin, a medication for diabetes management, appears to lower liver cancer risk, highlighting the significance of diabetes control in impacting its risk (31). A study indicated that metformin may be associated with a 62% decrease in the risk of liver cancer among individuals with type 2 diabetes (32). Therefore, diabetes regulation is a critical preventive measure against liver cancer, and further exploration of different other medications may be essential in this regard.

### 4.2 Liver cancer risk and social determinants of health

Our study delves into the impact of SDOHs, the non-medical conditions influencing health outcomes. Among these determinants, employment status, educational level, place of residence, and marital status emerge as significant factors associated with liver cancer risk.

Our findings indicate that unemployed individuals had 0.01 lower odds of being affected by liver cancer compared to their employed counterparts, suggesting that unemployment is a protective factor against liver cancer. Thus, being employed rather than unemployed could be associated with an increased odds of liver cancer.

Noting that the unemployed category encompasses students, homemakers, and individuals currently out of work, these often represent a younger age group in good health but facing joblessness due to their living conditions. In contrast, the retired participants face 4.5 times higher risks of developing liver cancer, compared to the employed group. This discrepancy may be partially attributed to the fact that the retired category predominantly includes individuals from an older age group. This prediction was supported by our supplementary analysis, presented in **Supplementary Table 1**, which showed that 55.83% of the unemployed participants fall in the younger age group (18 to 34 years), and about 83% of the retired participants are in the older age group, 65 or older. This aligns with a recent study that established an increase in the prevalence of certain liver cancer conditions with age, and an increased prevalence of advanced liver diseases among older individuals compared to their younger counterparts (55). Moreover, advanced age was found to be the primary determinant for complications and progression to end-stage liver cancer, as well as presenting challenges in the use of targeted therapeutic interventions in older adults (55).

The age distribution among the employment status may in part explain the detected association between employment and liver cancer risk. However, other factors can also contribute to the increase in the risk of cancer among previously employed and currently retired individuals, or among those who are currently employed. These factors include exposure to certain occupational carcinogens and hazardous materials such as asbestos, lead, pesticides, among other chemicals and substances, and sun exposure (56). Moreover, increased risk of cancer can also be induced by work-related stress and shift work especially the night shifts which can cause a disruption in certain hormone levels (57). For instance, a study has shown that full-time working women were at higher risk of having cancer than staying home women who were full-time caretakers of their households (12). Several explanations were proposed for these findings including social and psychological stressors, less time to health promoting behaviors, in addition to the exposure to hazardous environments (12, 58).

Another crucial SDOH significantly associated with liver cancer risk is the place of residence, particularly whether in a rural or urban area. Our study’s findings suggest that living in an urban area is a protective factor compared to rural areas. This protective effect may result from increased accessibility to healthcare resources in urban settings, facilitating regular checkups, immediate treatments, and efficient diagnosis in well-established hospitals (59). Our research aligns with the results of a Chinese study examining the incidence of liver cancer, which found that rural areas had a higher incidence rate compared to urban areas based on geographical analysis (60). Specifically, the crude incidence rates were 35.78 per 100,000 in rural areas and 21.64 per 100,000 in urban areas (60). Furthermore, after adjusting for age, the incidence rates remained higher in rural areas, with 34.34 per 100,000 in rural and 15.72 per 100,000 in urban areas (60). Moreover, in a nationwide study conducted from 2000 to 2016, targeting adults newly diagnosed with HCC, the focus was on disease manifestation and prognosis disparities across rural and urban regions (59). The findings showed that individuals residing in rural and suburban areas in the US were more likely to be diagnosed with HCC at a later stage and less likely to receive treatment (59). Additionally, the socioeconomic and geographic disparities were highlighted by a study of regional variations among patients with ICC in a Texas cancer registry (61). The analysis indicated that individuals residing in low-income regions were less likely to receive treatment, suggesting that residing in rural areas heightens liver cancer risk, leading to poorer outcomes and survival rates (61). Furthermore, environmental exposures may help explain the elevated liver cancer risk among rural residents. For example, a case-control study among California residents in agriculturally intensive areas found that exposure to organochlorine pesticides was associated with increased HCC risk, even after adjusting for liver disease and diabetes, particularly in men, with an odds ratio of 2.76 (95% CI: 1.58–4.82) (62). Similarly, a meta-analysis investigating HCC risk factors highlighted that agricultural work, common in rural settings, and pesticide exposure are significant contributors to HCC development. These findings emphasize the importance of educating rural populations on the safe use of pesticides and promoting access to lower-toxicity alternatives to improve health outcomes and reduce liver cancer risk (63).

Educational level emerged as a significant predictor for liver cancer in our study, with high school graduates and college graduates having seven-fold and five-fold higher risk, respectively, compared to those with some high school or below, the reference category. A possible explanation of this association may be reflective of the link between higher educational attainment, improved healthcare access, and regular health screenings, which can contribute to early detection and increase diagnosis rates (64). For instance, a study revealed that highly educated individuals are more likely to participate in cancer screening at their own initiative (65), a finding that suggests that better education fosters greater health awareness, potentially elevating cancer diagnosis rates (66). However, it is important to distinguish here between the true risk differences and detection-related disparities. In this regard, a study examining temporal trends in liver cancer mortality by educational attainment in the US over 15 years showed that the rise in liver cancer death rates has primarily affected individuals with lower levels of education (67). This finding may be attributed to lower detection rate and delayed diagnosis in the group of individuals with lower educational levels, leading to poorer prognosis and decreased chances of survival (68, 69).

Nevertheless, our findings are in contrast to a study examining the impact of socioeconomic status on HCC, which revealed that patients with lower levels of education experienced worse short-term and long-term outcomes, resulting in elevated HCC mortality rates (70). However, it is noteworthy that the less educated patients in the latter study were characterized as older, low-income individuals residing in rural areas, exhibiting a more advanced tumor burden, and receiving fewer curative treatments and regional therapies (70). These factors may explain the underlying increased risk of liver cancer associated with low educational attainment, thereby opposing the results observed in our study.

To further explore this elevated liver cancer risk among more educated individuals in our sample, we analyzed educational attainment in relation to the place of residence (rural vs. urban) as detailed in **Supplementary Table 2**. Among rural residents, the largest proportion (35.45%) were high school graduates, followed by individuals with some college education (32.45%) and college graduates (18.56%). This suggests that living in rural areas may partly explain the increased odds of liver cancer seen among more educated individuals in our dataset.

Marital status was also identified as a risk factor for liver cancer in our analysis, with widowed individuals having the highest risk, followed by those who never married, compared to those who were married. Consistent with our findings, a recent study of 4,933 participants concluded that marriage appears to be protective against liver cancer, with non-married individuals having an elevated risk (HR: 1.15) and a worse prognosis compared to married individuals (18). Moreover, survival outcomes for liver cancer patients have been found to be better among married individuals. For example, a retrospective study using population-based data revealed that married participants had a higher 5-year HCC cause-specific survival rate of 46.7%, compared to 37.8% for unmarried participants, with widowed individuals showing the poorest survival rate at 29.4% (19). These associations may be explained by the greater social and emotional support that married individuals tend to receive compared to their non-married counterparts (22). Moreover, unmarried individuals are at higher risk for psychological distress, which is linked to poorer cancer prognosis (17, 21, 71).

### 4.3 Liver cancer risk and smoking

Our study highlighted that the risk of liver cancer was approximately 3 times higher among former smokers compared to individuals who have never smoked, highlighting smoking as a significant and enduring predictor for liver cancer even after cessation. Our results are consistent with findings from a population-based case-control study conducted among men in the US, which identified a link between cigarette smoking and the likelihood of primary liver cancer development (72). However, the strength of the association in their findings was comparatively weaker than in ours, with a reported OR of 1.85 for former smokers compared to non-smokers. Furthermore, their study concluded that an increased duration of smoking and a higher number of packs smoked per day were associated with an increased liver cancer risk (72). Likewise, a recent prospective cohort study conducted in China reached a similar conclusion, indicating that former smokers exhibit an increased liver cancer risk compared to non-smokers. The study reported a multivariable-adjusted hazard ratio of 1.42 for former smokers in comparison to non-smokers, and a 49% elevated risk of liver cancer for individuals with a smoking history exceeding 40 years (73). Our dataset lacks information regarding the duration and intensity of smoking among participants. This lack of information encompasses individuals who may have recently quit smoking, posing a limitation in our study. A plausible explanation for the association between liver cancer and smoking is provided by a study indicating that carcinogenic compounds in cigarette smoke stimulate the progression of liver cancer by inducing inflammation and fibrosis (36, 74). For instance, N-nitrosodimethylamine is recognized for its role in promoting liver fibrosis and subsequent cancerous transformations. Similarly, 4-Aminobiphenyl undergoes metabolic breakdown within the liver, primarily facilitated by hepatic CYP1A2 enzymes (74). This compound forms DNA adducts, which are abnormal chemical structures created when a compound binds to DNA. These adducts can disrupt normal cellular processes, thus increasing the likelihood of cancer development within liver cells (74).

### 4.4 Limitations

This study provided valuable insights into liver cancer risk factors, including diabetes, specific SDOHs, biological and behavioral factors. However, several limitations warrant acknowledgment. Our analysis is based on data from the BRFSS, where information is self-reported and lacks objective diagnosis by healthcare professionals, potentially impacting data accuracy. Additionally, as mentioned previously, not all states responded to the cancer-related questions, leading to missing data from these states. Moreover, the lack of specific questions on diabetes medications, alcohol and smoking duration, type of occupation, exposure to occupation-related hazardous substances, as well as the type of liver cancer, presents additional limitations to our study. Additionally, the BRFSS does not collect data on key known risk factors for liver cancer, such as viral hepatitis B and C or non-alcoholic fatty liver disease (NAFLD), which could have provided valuable insights for our analysis. Furthermore, although our sub-analysis provided initial insights into the ages of diagnosis of diabetes and liver cancer and their potential correlation, the small number of liver cancer cases utilized in this sub-group analysis limited the robustness and generalizability of these findings, making it difficult to draw firm conclusions about diabetes as a risk factor.

## 5 Conclusions and implications

In this comprehensive study that explored potential risk factors for liver cancer, we found that diabetes was significantly associated with an increased risk of liver cancer, whilst adjusting for the effect of a diverse array of social, biological, and behavioral variables. These findings suggest that prioritizing diabetes prevention and management efforts may play an important role in reducing liver cancer risk at the population level.

Our study also identified a higher risk of liver cancer among rural residents, which may be explained by limited access to healthcare and resources in these areas. Furthermore, we observed increased liver cancer risk among certain demographic groups, including widowed or never married, employed or retired individuals, and those with higher education. These findings emphasize the importance of considering socioeconomic factors as part of SDOHs in our comprehension of liver cancer risk, which necessitates directing more attention toward these specific demographic groups to address the underlying disparities effectively.

In conclusion, this study offers valuable preliminary insights into the range of factors potentially associated with liver cancer risk. However, confirming these associations as true risk factors will require future cohort studies with adequate number of liver cancer cases, comprehensive data collection, and careful attention to the limitations outlined in this study. Such research is essential to strengthen the evidence base and guide effective prevention and intervention efforts.

## Data Availability

Publicly available datasets were analyzed in this study. This data can be found here: https://www.cdc.gov/brfss/annual_data/annual_2022.html.

## Supplementary material

Supplementary Table 1 Weighted percentage (row percent) of the distribution of the participants based on their age and their employment status.

Supplementary Table 2 Weighted percentage (column percent) of the distribution of the participants based on their educational level and their place of residence.
