# Supplementary material for: The impact of diabetes and social, biologic and behavioral determinants of health on liver cancer risk
Source: Front Endocrinol (Lausanne). 2025 Jun 27;16:1562854. doi: 10.3389/fendo.2025.1562854 (PMC12245665; doi:10.3389/fendo.2025.1562854)
Supplement: Supplementary Table 1 — Weighted percentage (row percent) of the distribution of the participants based on their age and their employment status. [file Table1.docx]

Supplementary Table 1: Weighted percentage (row percent) of the distribution of the participants based on their age and their employment status.

|  | Age Categories | | | | P-value: <0.001 |
| --- | --- | --- | --- | --- | --- |
| Employment Status | 18 to 34 | 35 to 54 | 55 to 64 | 65 or older |  |
| Employed | 34.68% | 42.18% | 16.68% | 6.46% |  |
| Unemployed | 55.83% | 26.51% | 9.92% | 7.74% |  |
| Retired | 0.31% | 3.29% | 13.63% | 82.77% |  |
| Unable to work | 13.03% | 33.48% | 36.23% | 17.26% |  |
