# Supplementary material for: The impact of diabetes and social, biologic and behavioral determinants of health on liver cancer risk
Source: Front Endocrinol (Lausanne). 2025 Jun 27;16:1562854. doi: 10.3389/fendo.2025.1562854 (PMC12245665; doi:10.3389/fendo.2025.1562854)
Supplement: Supplementary Table 2 — Weighted percentage (column percent) of the distribution of the participants based on their educational level and their place of residence. [file Table2.docx]

Supplementary Table 2: Weighted percentage (column percent) of the distribution of the participants based on their educational level and their place of residence.

| Educational level | Rural | Urban | p-value: <0.001 |
| --- | --- | --- | --- |
| Some high school or below | 13.58% | 9.61% |  |
| High school | 35.4% | 27.52% |  |
| Some college | 32.45% | 30.58% |  |
| College graduates | 18.56% | 32.29% |  |
